# Expansion and Nitrate-Responsive Expression of NRT3 Transport Regulators in Maritime Pine

**DOI:** 10.3390/plants15172690

**Published:** 2026-09-02

**Authors:** José Miguel Valderrama-Martín, Francisco Ortigosa, Concepción Ávila, Francisco M. Cánovas, Rafael A. Cañas

**Affiliations:** 1 Centre for Research in Agricultural Genomics (CRAG), CSIC-IRTA-UAB-UB, 08193 Barcelona, Spain; 2 Grupo de Biología Molecular y Biotecnología, Departamento de Biología Molecular y Bioquímica, Universidad de Málaga, Campus Universitario de Teatinos, 29010 Málaga, Spain; fortigosa.academic@hotmail.com (F.O.); cavila@uma.es (C.Á.); canovas@uma.es (F.M.C.); 3 Integrative Molecular Biology Lab, Universidad de Málaga, Campus Universitario de Teatinos, 29010 Málaga, Spain; rcanas@uma.es

**Keywords:** conifers, nitrate transport, *NRT3*, *NPF*, *NRT2*, gene expansion

## Abstract

Nitrate uptake in plants is mediated by coordinated transporter systems, which include NPF, NRT2 and NRT3 proteins. While these families have been extensively studied in angiosperms, their evolution and regulation in conifers are still not well understood. In this work, we examined the NRT3 family in maritime pine (*Pinus pinaster*) and in representative plant lineages. Phylogenetic analyses of nucleotide sequences and the NRT3 protein revealed a broad expansion in gymnosperms, particularly in conifers, while copy number increases among angiosperms appeared to be more lineage-specific. In addition, we evaluated the expression of nitrate transporter genes in cotyledons, hypocotyls and roots of *P. pinaster* seedlings exposed to low and high concentrations of nitrate. Several *NRT3* genes, particularly *PpNRT3.1*, *PpNRT3.3* and *PpNRT3.5*, were significantly induced by nitrate, while most *NPF* and *NRT2* genes showed weaker or non-significant transcriptional responses. Correlation analysis revealed distinct expression associations among the NRT3, NRT2 and NPF transporters, including a specific association between *PpNRT3.4* and *PpNRT2.1*, as well as broader correlations among other *NRT3* paralogs and *NPF* genes. These results indicate that the expansion of *NRT3* in conifers was accompanied by transcriptional divergence among paralogs and identify potential regulatory relationships for future functional studies.

## 1. Introduction

Nitrate is both a major nitrogen source and a signaling molecule that regulates plant metabolism and development [1,2,3,4]. Nitrate uptake and transport are mediated by three main families of transporters: NPF (nitrate transporter family 1/peptide transporter family), NRT2 (nitrate transporter family 2), and NRT3 (nitrate transporter family 3) [5]. The NRT3 family is particularly interesting because it functions as an associate protein that modulates the high-affinity transport activity of NRT2 [6,7], and studies show enhanced NRT2 activity when co-expressed with NRT3 [4,8,9].

Although conifers have traditionally been considered preferential users of ammonium, recent evidence confirmed that *Pinus pinaster* can efficiently acquire and assimilate nitrate [10,11,12]. Notably, Castro-Rodríguez et al. (2017) [13] identified an unusual expansion of the NRT3 family in maritime pine, with 5–6 members compared to only 1–2 in angiosperms, raising the hypothesis that NRT3 expansion may have been accompanied by functional diversification in gymnosperms. However, the functions of the individual paralogs remain unresolved.

This study combined phylogenetic analysis with nitrate-responsive gene expression profiling to investigate the evolutionary and regulatory diversification of the NRT3 family. It explored whether the NRT3 expansion is conserved across conifer lineages and whether individual *NRT3* genes in *P. pinaster* exhibit distinct transcriptional responses to nitrate. Furthermore, expression correlations between *NRT3*, *NRT2*, *NPF* and nitrate assimilation genes were analyzed to identify candidate regulatory modules associated with nitrate nutrition in pine.

## 2. Results

### 2.1. Phylogenetic Analyses

Castro-Rodríguez et al. (2017) [13] previously characterized nitrate transporters in *P. pinaster* and reported an expansion of the NRT3 family in maritime pine with six members. However, a re-examination of the sequences indicated that *PpNRT3.5* is most likely an allelic variant of *PpNRT3.4*. The two predicted proteins comprised 215 amino acids, shared 96.7% sequence identity and differed at only seven positions (Figure S2A, in Supplementary Material S5). Alignment of the full-length transcripts also showed extensive nucleotide conservation across the overlapping regions (Figure S2B, in Supplementary Material S5). Moreover, only a single *PpNRT3.4*-like region was identified in the available *P. pinaster* genome, while only *PpNRT3.4* could be cloned from cDNA. No independent genomic locus or cloned transcript supporting the former *PpNRT3.5* as a separate gene was detect*ed.* Nevertheless, because the available genomic sequence is incomplete, the possibility that these sequences originated from a very recent gene duplication cannot be formally excluded. Therefore, *PpNRT3.6* has been renamed *PpNRT3.5* in the present work. Interestingly, compared to angiosperm NRT3 families, which are generally composed of two members, most gymnosperm NRT3 families were expanded, presenting up to five members.

**Figure 1:**
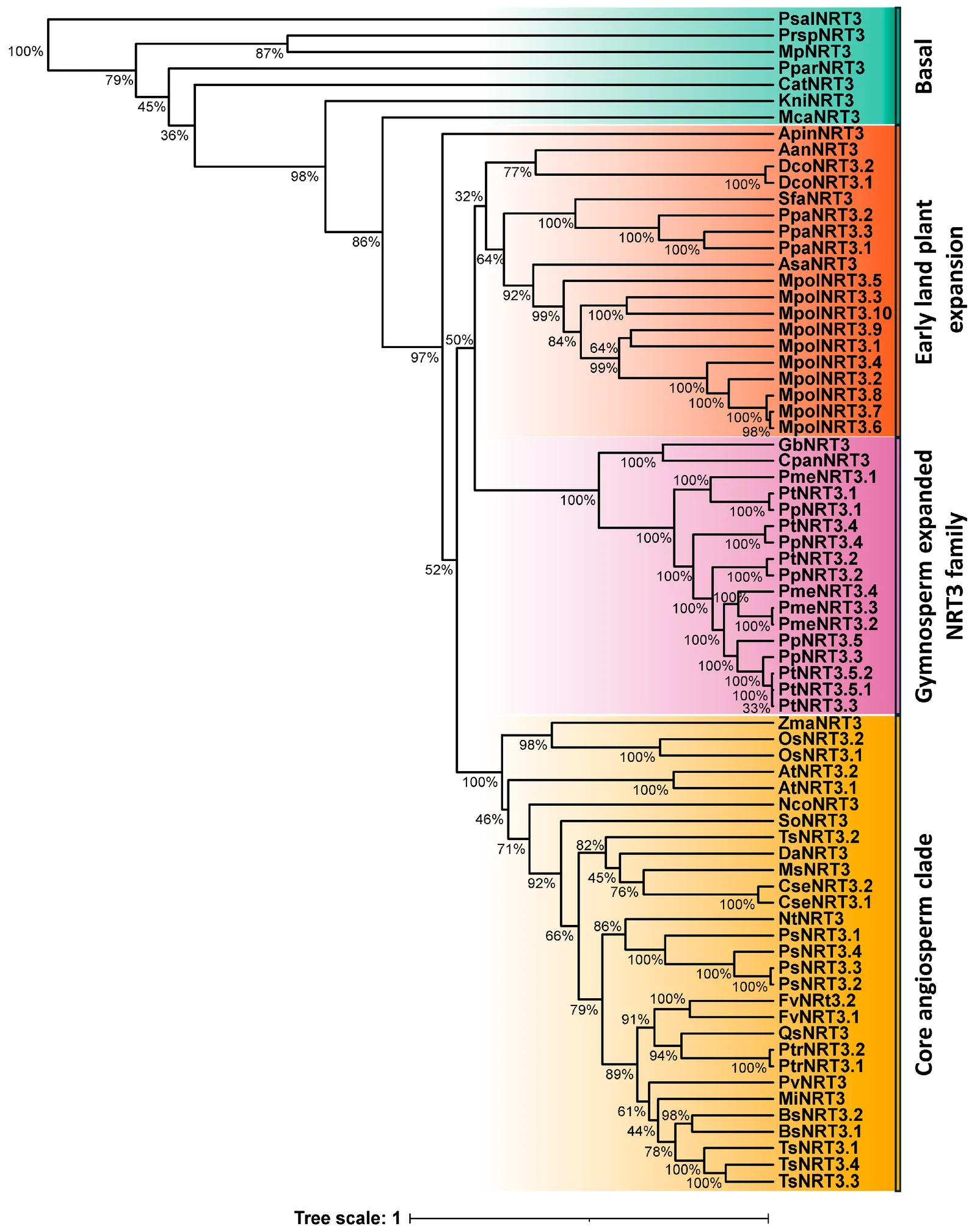
Bayesian phylogenetic analysis of the NRT3 protein family in plants. The maximum credibility clade tree was inferred from NRT3 protein sequences using BEAST2 with the WAG amino acid substitution model with gamma-distributed rate heterogeneity, a strict molecular clock and a Yule tree prior distribution. *Picocystis salinarum* was used as outgroup. Node values indicate Bayesian posterior probabilities expressed as percentages. The main NRT3 groups are highlighted according to their phylogenetic distribution, including early-diverging/basal homologs, expanded lineages in early land plants, expanded NRT3 paralogs in gymnosperms/conifers and angiosperm-associated clades. Species abbreviations: Aan, *Anthoceros angustus*; Apin, *Azolla pinnata*; Asa, *Acrolejeunea sandvicensis*; At, *Arabidopsis thaliana*; Bs, *Buxus sempervirens*; Cat, *Chlorokybus atmophyticus*; Cpan, *Cycas panzhihuaensis*; Cse, *Chloranthus sessilifolius*; Da, *Dioscorea alata*; Dco, *Diphasiastrum complanatum*; Fv, *Fragaria vesca*; Gb, *Ginkgo biloba*; Kni, *Klebsormidium nitens*; Mca, *Mougeotiopsis calospora*; Mi, *Macadamia integrifolia*; Mp, *Micromonas pusilla*; Mpol, *Marchantia polymorpha*; Ms, *Magnolia sinica*; Nco, *Nymphaea colorata*; Nt, *Nicotiana tabacum*; Os, *Oryza sativa*; Ppa, *Physcomitrium patens*; Ppar, *Pyramimonas parkeae*; Pme, *Pseudotsuga menziesii*; Pp, *Pinus pinaster*; Prsp, Prasinococcaceae sp. CCMP; Ps, *Papaver somniferum*; Psal, *Picocystis salinarum*; Pt, *Pinus taeda*; Ptr, *Populus trichocarpa*; Pv, *Phaseolus vulgaris*; Qs, *Quercus suber*; Sfa, *Sphagnum fallax*; So, *Spinacia oleracea*; Ts, *Tetracentron sinense*; Zma, *Zostera marina*.

Bayesian phylogenetic analyses revealed marked variation in NRT3 copy number among plant lineages (Figure 1). Nucleotide-based analysis recovered a broadly comparable phylogenetic structure, although some internal relationships differed between datasets (Figure S1 in Supplementary Material S5). Based on protein phylogeny, early-diverging algal representatives generally contained a single NRT3 homolog, whereas expansions were already evident in some early land plants, including *Marchantia polymorpha* and *Physcomitrium patens*. In seed plants, gymnosperm NRT3 sequences were distributed among several well-supported paralogous groups containing representatives in multiple conifer species. In contrast, NRT3 copy number increases among angiosperms appeared more restricted to certain lineages, including *Papaver somniferum* and *Tetracentron sinense*.

### 2.2. Gene Expression Analysis

**Figure 2:**
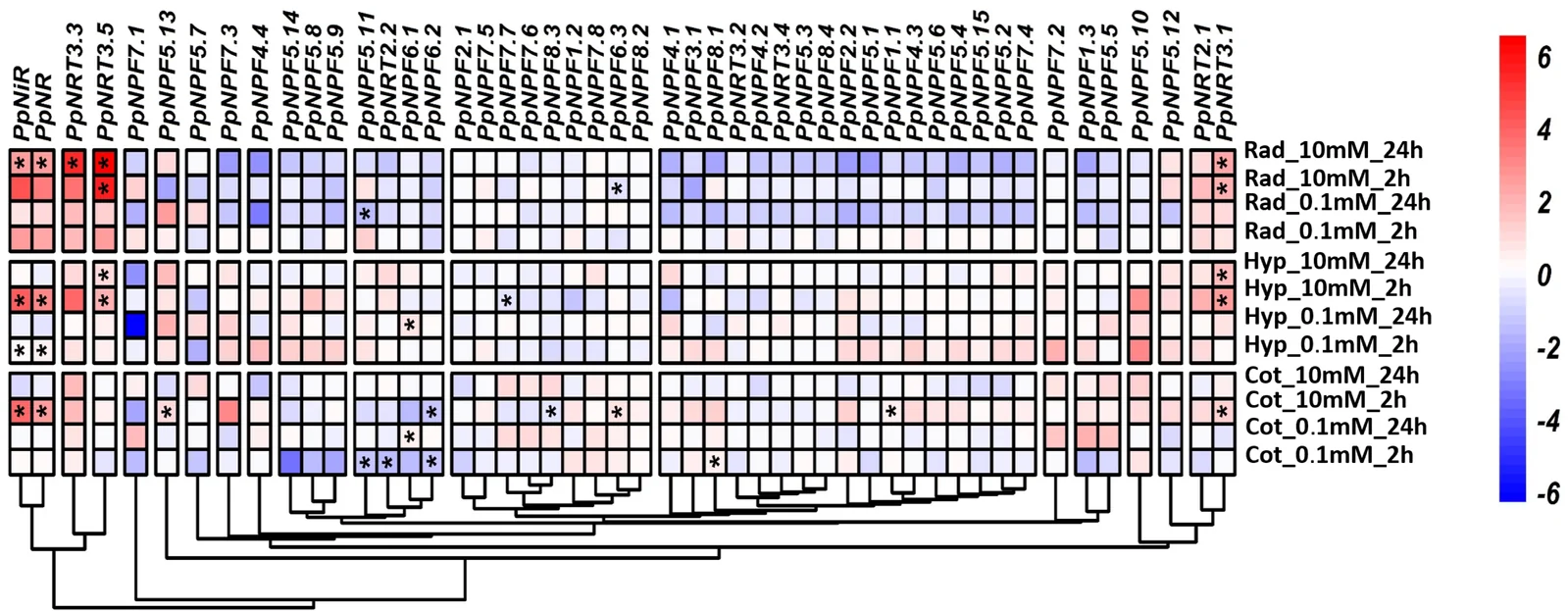
Heatmap of expression data for the NPF, NRT2 and NRT3 transporters from *P. pinaster* obtained by RT-qPCR. Expression values are presented as log_2_ fold-changes relative to the corresponding water-treated control from the same tissue and sampling time. Genes with significant upregulation or downregulation (*t*-test, *p* < 0.05) are marked with an asterisk.

To determine whether NRT3 expansion is associated with nitrate-responsive expression, we examined the transcriptional response of nitrate transporter genes in cotyledons, hypocotyls and roots of *P. pinaster* seedlings treated with 0.1 or 10 mM KNO_3_ and harvested after 2 or 24 h (Figure 2). The genes encoding nitrate reductase (*PpNR*) and nitrite reductase (*PpNiR*) were included as markers of the primary nitrate response. Both genes were strongly induced after nitrate supply, particularly in seedlings treated with 10 mM nitrate for 2 h, confirming that the experimental treatments triggered a clear transcriptional response to nitrate (Figure 2, *PpNR*, *PpNiR*).

Among the nitrate transporters examined, only eight genes (*PpNRT3.1*, *PpNRT3.3*, *PpNRT3.5*, *PpNPF1.1*, *PpNPF6.1*, *PpNPF6.3*, *PpNPF5.13*, and *PpNPF8.1*) showed significant upregulation, predominantly in tissues exposed to 10 mM nitrate at either 2 h or 24 h post-treatment. Notably, three of the five NRT3 members were significantly induced, whereas neither *PpNRT2.1* nor *PpNRT2.2* showed significant upregulation.

Most *NPF* genes showed weak or non-significant responses, although significant repression was detected for a limited number of members. *PpNPF6.2* was downregulated in cotyledons after 2 h at both nitrate concentrations, *PpNPF5.11* was repressed in cotyledons and roots at low nitrate, and *PpNPF8.3* was downregulated in cotyledons after 2 h at 10 mM nitrate. *PpNPF6.3* showed an organ-dependent response, being downregulated in roots but induced in cotyledons after 2 h at 10 mM nitrate.

### 2.3. NRT3 Paralogs Display Distinct Co-Expression Patterns

**Figure 3:**
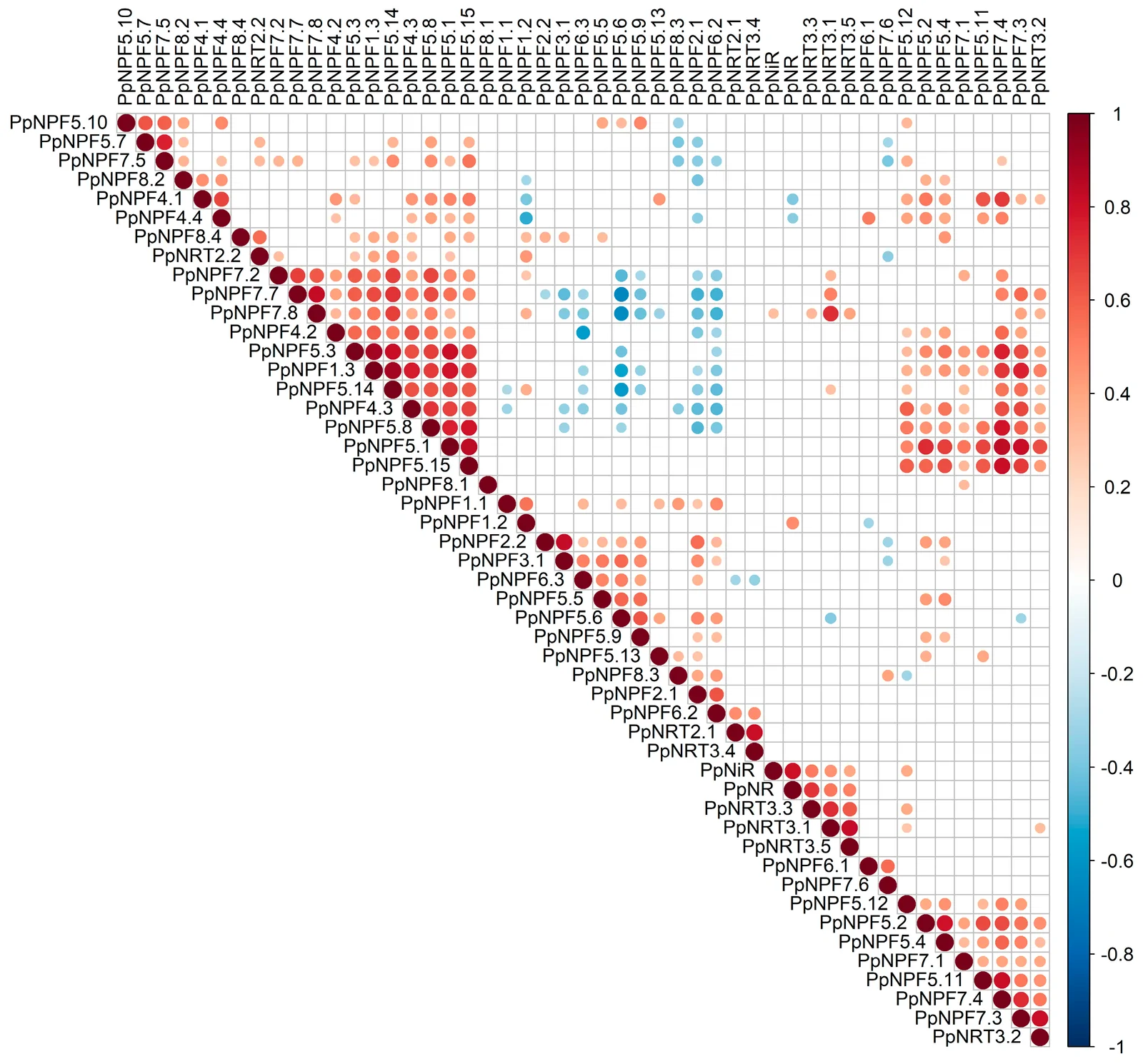
Correlation map with nitrate transporter expression data. Only significant Pearson correlations (*p* < 0.01) are shown with dots. The size and color intensity of the dots indicate the correlation coefficients. Red dots show positive correlations. Blue dots represent negative correlations.

A correlation matrix was constructed from these gene expression data (Figure 3) to identify coordinated expression patterns among putative nitrate transporters. Of the two NRT2 members, only *PpNRT2.1* was significantly correlated with an *NRT3* gene, specifically *PpNRT3.4*. Simultaneously, *PpNRT3.4* expression showed correlations with the expression of both *PpNPF6.2* (positive correlation) and *PpNPF6.3* (negative correlation). Interestingly, *PpNRT2.1* also showed a negative correlation with *PpNPF6.3*, and neither *PpNRT3.4* nor *PpNRT2.1* were significantly correlated with *PpNR* and *PpNiR*.

The remaining *NRT3* paralogs showed contrasting association patterns. *PpNRT3.1*, *PpNRT3.3* and *PpNRT3.5* were positively correlated with *PpNR* and *PpNiR*, consistent with their nitrate-responsive expression. *PpNRT3.1* was also associated with several *NPF5* and *NPF7* genes, while *PpNRT3.2* showed the broadest association with the NPF family, including members of the NPF1, NPF4, NPF5 and NPF7 subfamilies. In contrast, *PpNRT3.5* correlated with other nitrate-responsive NRT3 members, but not with the analyzed *NPF* genes. *PpNPF7.8* was particularly noteworthy because it was positively correlated with all *NRT3* members except *PpNRT3.4*.

## 3. Discussion

### 3.1. Evolutionary Diversification and Retention of NRT3 Paralogs in Conifers

Phylogenetic analysis conducted in this work revealed an expansion of the NRT3 family of proteins in different plant lineages, which might represent an attempt to diversify the use of soil nitrogen resources, associated with a more efficient ecological strategy for land colonization.

A subsequent expansion of the family occurred in the gymnosperms, exclusively in conifers. Interestingly, this clustering, along with the high posterior probability values, suggests that the expansion observed in gymnosperms occurred in a common ancestor of this plant group, rather than through independent, lineage-specific duplications in individual genera.

Previous phylogenomic analysis also detected an expansion of the NRT3 family in gymnosperms [14], while a recent genome-wide analysis of *Pinus koraiensis* identified multiple *NRT3* genes [15]. These results are consistent with the existence of ancient whole-genome duplications (WGD) at the base of all seed plants [16,17]. However, the absence of later genome duplication events suggests high genomic stability in conifers, in contrast to that found in angiosperms where multiple WGD correlate with evolutionary diversification and major impact on gene novelty [18,19].

Genetic duplication is a widespread phenomenon in plants [19] that can facilitate functional diversification, thus opening the possibility of new strategies that allow adaptation to new environmental niches. Interestingly, this expansion of NRT3 in gymnosperms is not observed in the NRT2 transporter family, which maintains a number of members comparable to that of angiosperms, despite the established role of NRT3 proteins in modulating NRT2 activity [4,8,9].

The contrasting evolutionary patterns of the NRT3 and NRT2 families raise questions about the functional significance of NRT3 expansion in conifers. While NRT3 proteins have previously been shown to enhance the activity of AtNRT2.1 and AtNRT2.2 [8,9], the distinct pattern observed here, along with data presented by Ortigosa et al. (2020) [10], could reflect the different ecological strategies and nutritional requirements of conifers compared to angiosperms. This interpretation aligns with the traditional notion that conifers generally show a preference for ammonium over nitrate [20,21,22,23,24,25,26], whereas an expansion of the NRT3 family may provide regulatory flexibility for nitrate utilization under conditions where ammonium is limiting. Although conifers are predominantly distributed in boreal ecosystems where nitrification occurs at lower rates due to low temperatures [27], some, including *P. pinaster*, are native to temperate climate ecosystems that permit nitrification to take place. The plant material used in this study comes from the Western Mediterranean region, where such conditions prevail. Ortigosa et al. (2020) [10] demonstrated that, despite increased biomass production with ammonium nutrition, maritime pine retained the ability to utilize nitrate. Similarly, Zhou et al. (2021) [11] documented the efficient use of both ammonium and nitrate by four conifer species in their mature state within their natural environments. However, nitrate uptake capacity may change throughout tree development, as nitrogen acquisition strategies in mature trees can shift with tree age in association with changes in fine-root functional traits and ectomycorrhizal associations [28,29]. Therefore, the physiological and ecological differences between seedlings and mature trees should be considered when interpreting nitrate responses, and results obtained in controlled seedling systems should be extrapolated to mature trees under field conditions only with caution.

### 3.2. Selective Nitrate Responsiveness and Distinct Co-Expression Patterns Among Nitrate Transporter Genes

The expression analysis was designed to determine whether nitrate elicited an early transcriptional response at 2 h and whether this response was maintained or modified after 24 h. The strong induction of *PpNR* and *PpNiR* at 2 h confirmed that the experiment captured a clear primary transcriptional response to nitrate. However, Ortigosa et al. (2020) [10] demonstrated that nitrate uptake in *P. pinaster* peaked approximately 15 min after nitrate application and declined sharply by 30 min. These rapid uptake kinetics provide a plausible explanation for the limited transcriptional response of most *NRT2* and *NPF* genes at 2 h: transient changes in transporter expression may have occurred before the first sampling point, or transporter activity may have been regulated without sustained changes in transcript abundance. Accordingly, the induction of several *NRT3* genes without sustained upregulation of *NRT2* transcripts does not exclude regulation downstream of transcription. In *Arabidopsis thaliana*, high-affinity nitrate uptake is controlled through changes in NRT2.1/NAR2.1 protein abundance and through phosphorylation-dependent modulation of NRT2.1 activity, stability and interaction with NAR2.1 [30,31,32,33]. A similar mechanism could operate in *P. pinaster*, although this possibility was not directly examined in the present study.

Indeed, the specific association between *PpNRT3.4* and *PpNRT2.1* is consistent with a canonical NRT2–NRT3 expression module and raises the possibility that their encoded proteins interact, as demonstrated for Arabidopsis NRT2 transporters and AtNAR2.1/AtNRT3.1 [8,9]. However, the broader correlation patterns cannot be fully explained by a simple one-to-one NRT2–NRT3 co-expression model and raise the hypothesis that some conifer NRT3 proteins may have roles beyond NRT2 modulation. The associations with members of the NPF family provide candidate relationships for investigating this hypothesis, particularly because NPF proteins transport nitrate together with a wide variety of other molecules [4], while nitrate itself acts as a signalling molecule that regulates different biological processes [3]. Nevertheless, this interpretation remains speculative: these correlations may reflect shared responses to tissue identity or treatment and do not demonstrate direct regulatory or physical interactions. Therefore, the correlation matrix should be considered a hypothesis-generating tool rather than evidence of interactions between NRT3 and NPF proteins.

Several limitations must be considered. First, the expression analysis was performed on young seedlings, whose root architecture, nitrogen requirements, and nutrient allocation patterns differ from those of adult trees. Moreover, the controlled experimental system does not reproduce the complexity associated with soil nitrogen availability, fine-root structure and mycorrhizal interactions under field conditions. Therefore, the expression patterns observed here should be interpreted specifically as acute responses of juvenile *P. pinaster* plants to nitrate supply under controlled conditions and should not be extrapolated directly to nitrate uptake or long-term nitrogen acquisition strategies in mature trees. Nevertheless, this limitation does not affect the phylogenetic evidence for NRT3 family expansion, which is independent of the developmental stage used for the expression analysis and applies to the conifer lineages included in the comparative analysis. Second, gene expression analysis over time, at earlier stages following nitrate irrigation, would be necessary to fully characterize the transcriptional dynamics of these transporters. Third, co-expression does not demonstrate direct physical or regulatory interactions. Codon-based molecular evolutionary analyses will be required to assess whether individual *NRT3* paralogs show signatures of selection, while heterologous transport assays, protein interaction analyses and functional characterization will be needed to determine whether these paralogs have functionally diverged and to establish their specific functions.

## 4. Materials and Methods

### 4.1. Phylogenetic Analysis of NRT3 Family

The phylogeny of the NRT3 transporter family in plants was analyzed using sequences from a wide range of plant species, including green algae, non-vascular land plants, vascular plants, gymnosperms, and angiosperms. The nucleotide and protein sequences used in these analyses were obtained from Phytozome [34] and GenBank [35] using TBLASTN and BLASTP from NCBI BLAST+ version 2.17.0, with NRT3 sequences from *P. pinaster* as the initial query. The final dataset consisted of 72 NRT3 protein sequences and 72 nucleotide sequences. Alignment was conducted using MUSCLE in MEGA version 12 [36,37]. Sequence metadata and FASTA files for proteins and nucleotides are provided in Supplementary Material S1.

Bayesian phylogenetic analyses were performed in BEAST2 version 2.7.8 [38]. Protein sequences were analyzed using the WAG amino acid substitution model with gamma-distributed rate heterogeneity, while nucleotide sequences were analyzed using the GTR model with gamma-distributed rate heterogeneity. A strict molecular clock and a Yule tree a priori distribution were used in both analyses. Markov chain Monte Carlo analyses were run for 10,000,000 generations, with trees sampled every 1000 generations. Convergence and effective sample sizes were assessed using Tracer version 1.7.2. Maximum clade credibility trees were generated with TreeAnnotator version 2.7.8 after discarding the first 10% of samples as burn-in. Because temporal calibration or tip dates were not included, branch lengths were not interpreted as divergence times. *Picocystis salinarum* was used as the outgroup. Additional details of sequence retrieval, alignment generation and phylogenetic analysis are provided in Supplementary Material S2.

### 4.2. Plant Material and Nitrate Treatments

Maritime pine seeds (*P. pinaster* Aiton) from “Sierra Segura y Alcaraz” (EAN/055/10, Albacete, Spain) were provided by the “Red de Centros Nacionales de Recursos Genéticos Forestales of the Spanish Ministerio para la Transición Ecológica y el Reto Demográfico” with authorization number ESNC87. The seeds were germinated as described in Cañas et al. (2006) [39]. The germinated pines were grown in vermiculite in plant growth chambers (Fitoclima 1200; Aralab, Rio de Mouro, Portugal) under a 16 h light photoperiod, 125 μmol m^−2^ s^−1^ light intensity, 50% relative humidity and 23 °C. After one month, the seedlings were transferred to forest seedbeds and watered with a nitrogen-free nutrient solution. This period was included to minimize transient transcriptional responses associated with transplantation and handling. Nitrogen was omitted to avoid prior exposure to the experimental stimulus and to establish a common nutritional background before nitrate application. After three days of acclimation, the seedlings were treated with water, 0.1 mM KNO_3_ or 10 mM KNO_3_. Samples were taken before treatment and at 2 and 24 h after watering, separating them into cotyledons, hypocotyls and roots. For each replicate, tissue samples from six to seven seedlings were pooled, immediately frozen in liquid nitrogen and stored until RNA extraction. The experiment consisted of three independent replicates. The complete composition of the nitrogen-free nutrient solution and further details of plant growth and nitrate treatments can be found in Supplementary Material S2.

### 4.3. Total RNA Extraction and RT-qPCR

Total RNA was extracted following Canales et al. (2012) [40]. RNA concentration and purity were assessed using a NanoDrop ND-1000 spectrophotometer (Thermo Fisher Scientific, Waltham, MA, USA), and RNA integrity was verified by electrophoresis. Reverse transcription was performed with 500 ng of total RNA using iScript Reverse Transcription Supermix (Bio-Rad Laboratories, Hercules, CA, USA). RT-qPCR reactions were conducted with three biological and three technical replicates per tissue and condition using SsoFast EvaGreen Supermix (Bio-Rad Laboratories, Hercules, CA, USA) on a C1000 Thermal Cycler equipped with a CFX384 Touch Real-Time PCR Detection System (Bio-Rad Laboratories, Hercules, CA, USA). Expression values were normalized using saposin-like aspartyl protease and RNA-binding protein as reference genes [41].

Relative expression was analyzed with the qpcR package version 1.4-2 using the MAK3 model [42] in R version 4.6.1. Expression profiles were plotted as log_2_ fold-changes calculated by comparing each nitrate-treated sample with the corresponding water-treated control from the same tissue and sampling time. Significant expression differences were determined with a *t*-test (*p*-value < 0.05) and heatmaps were then generated with the pheatmap package version 1.0.13 [43]. Pearson correlation coefficients were calculated from the complete expression dataset (*n* = 54) and visualized using corrplot version 0.95 [44]. The detailed reaction composition, amplification conditions and melting curve analysis are described in Supplementary Material S2. Primer sequences are provided in Supplementary Material S3. Complete results of gene expression, including tissue, nitrate concentration, sampling time, log_2_ fold-change values and statistical significance, are provided in Supplementary Material S4. Complete pairwise Pearson correlation coefficients, associated *p*-values and sample sizes are provided in Supplementary Material S4.

## 5. Conclusions

In conclusion, the NRT3 family has undergone evolutionary expansion in conifers, and several *NRT3* genes in *P. pinaster* respond significantly to nitrate supply. The distinct transcriptional associations of individual paralogs with *NRT2*, *NPF* and nitrate assimilation genes support the hypothesis that the expansion of NRT3 was accompanied by regulatory diversification. These results lay the groundwork for experimentally evaluating the contribution of individual *NRT3* paralogs to nitrate nutrition in conifer trees.

## Data Availability

The datasets supporting the findings of this study are provided in the article and its Supplementary Information. Sequence metadata and protein and nucleotide FASTA files are provided in Supplementary Material S1. Detailed phylogenetic and experimental procedures are provided in Supplementary Material S2, and RT-qPCR primer sequences are provided in Supplementary Material S3. Complete nitrate-responsive expression and Pearson correlation results are provided in Supplementary Material S4. The nucleotide-based phylogenetic tree and the sequence alignments supporting the reassessment of *PpNRT3.4* and the former *PpNRT3.5* are presented as Figures S1 and S2, respectively, in Supplementary Material S5. Additional information is available from the corresponding authors on reasonable request.

## Supplementary Materials

The following supporting information can be downloaded at: https://www.mdpi.com/article/10.3390/plants15172690/s1, Supplementary Material S1: Sequence metadata and NRT3 sequence dataset, including the final list of species and sequence identifiers and the protein and nucleotide FASTA files used in the phylogenetic analyses; Supplementary Material S2: Detailed phylogenetic and experimental procedures, including sequence retrieval strategy, Bayesian phylogenetic settings, plant growth conditions, nutrient solution composition, nitrate treatments and RT-qPCR conditions; Supplementary Material S3. Complete list of primers used for RT-qPCR analysis, including target gene, primer name, nucleotide sequence and primer orientation; Supplementary Material S4. Complete nitrate-responsive gene expression and correlation results, including log_2_ fold-change values, Pearson correlation coefficients and associated *p* values; Supplementary Material S5. Nucleotide-based Bayesian phylogenetic analysis of the plant NRT3 family and sequence comparison between *PpNRT3.4* and the former *PpNRT3.5*. Figure S1 presents the maximum clade credibility tree inferred from the NRT3 nucleotide sequence dataset. Figure S2 presents amino acid and nucleotide alignments supporting the interpretation of the former *PpNRT3.5* sequence as an allelic variant of *PpNRT3.4*. References [45,46] are cited in the supplementary materials.
