# Supplementary material for: Expansion and Nitrate-Responsive Expression of NRT3 Transport Regulators in Maritime Pine
Source: Plants (Basel). 2026 Sep 2;15(17):2690. doi: 10.3390/plants15172690 (PMC13567595; doi:10.3390/plants15172690)
Supplement: Supplementary file 1 [file plants-15-02690-s001.zip › Supplementary_material_S2.pdf]

Supplementary Information

Supplementary Material S2. Detailed phylogenetic and experimental procedures

Supplementary Methods

S1. Sequence retrieval and Bayesian phylogenetic analysis

NRT3 protein and nucleotide sequences were retrieved from Phytozome (Goodstein et al. 2012) and GenBank (Clark et al. 2016) using maritime pine (*Pinus pinaster*) NRT3 sequences as initial queries. TBLASTN and BLASTP searches were performed across green algae, non-vascular land plants, vascular plants, gymnosperms, and angiosperms. Because sequence conservation was low among distantly related lineages, representative sequences recovered from each major phylogenetic group were subsequently used as additional queries. The final dataset comprised 72 NRT3 protein sequences and their corresponding 72 nucleotide sequences. Multiple sequence alignments were generated with MUSCLE (Edgar 2004) in MEGA12 (Kumar et al. 2024).

Protein and nucleotide alignments were analyzed independently in BEAST2 (Bouckaert et al. 2019). The protein dataset was analyzed using the WAG amino-acid substitution model with gamma-distributed rate heterogeneity, whereas the nucleotide dataset was analyzed using the GTR model with gamma-distributed rate heterogeneity. Tip dates were disabled, a strict molecular clock was applied, and a Yule tree prior was used in both analyses. Markov chain Monte Carlo analyses were run for 10,000,000 generations, sampling every 1,000 generations. Convergence and effective sample size values were evaluated in Tracer. Posterior tree distributions were summarized in TreeAnnotator after discarding the first 10% of samples as burn-in, and maximum clade credibility trees were generated using common-ancestor node heights. *Picocystis salinarum* was used as the outgroup. Because no temporal calibration or tip dates were included, branch lengths and node heights were not interpreted as divergence times.

To specifically assess the relationship between PpNRT3.4 and the former PpNRT3.5, their predicted protein sequences were aligned using the MultAlin web server version 5.4.1 (http://multalin.toulouse.inra.fr/multalin/; accessed on 26 August 2026) with the Identity-1-0 scoring scheme (Corpet 1988). The corresponding full-length transcript sequences were also aligned with the available PpNRT3.4 genomic region and coding sequence using the same settings. Amino acid identity was calculated over the complete 215-residue alignment, and divergent positions were identified from the resulting alignment. Protein and nucleotide comparisons are presented in Figure S2 in Supplementary Material 5.

S2. Plant growth, nutrient solution, and nitrate treatments

Maritime pine (*P. pinaster* Aiton) seeds from the "Sierra Segura y Alcaraz" provenance (EAN/055/10, Albacete, Spain) were provided by the Red de Centros Nacionales de Recursos Genéticos Forestales of the Spanish Ministerio para la Transición Ecológica y el Reto Demográfico under authorization ESNC87. Seeds were germinated as described by Cañas et al. (2006). Seedlings were grown in vermiculite in controlled-environment chambers under a 16 h photoperiod, a light intensity of 125 μmol m^{-2} s^{-1}, 50% relative humidity, and a constant temperature of 23 °C. During the first month, seedlings were irrigated twice weekly with water.

After one month, seedlings were randomly divided into three groups, transferred to forest seedbeds, and supplied with a nitrogen-free macro- and micronutrient solution containing 1.16 mM KCl, 0.63 mM KH_2PO_4, 0.35 mM $MgSO_4·7H_2O$, 0.17 mM $CaCl_2·H_2O$, 80 μM EDTA–$FeSO_4$, 25.9 μM H_3BO_3, 10.2 μM $MnCl_2·4H_2O$, 1.3 μM $ZnSO_4·7H_2O$, 0.7 μM $CuSO_4·5H_2O$, and 0.1 μM $Na_2MoO_4·2H_2O$. After three days of acclimation, control plants received 80 mL of water, whereas the two nitrate-treated groups received 80 mL of either 0.1 mM KNO_3 or 10 mM KNO_3. Samples were collected immediately before irrigation (0 h) and at 2 and 24 h after treatment. Cotyledons, hypocotyls, and roots were collected separately. For each biological replicate, tissues from six to seven seedlings were pooled, immediately frozen in liquid nitrogen, and stored until RNA extraction. The experiment was performed independently three times.

S3. RNA extraction, reverse transcription, and RT-qPCR

Total RNA was extracted following Canales et al. (2012). RNA concentration and purity were determined using a NanoDrop ND-1000 spectrophotometer (Thermo Fisher Scientific, Waltham, MA, USA), and purity was assessed from the A_{260}/A_{280} and A_{260}/A_{230} absorbance ratios. RNA integrity was checked by electrophoresis.

Reverse transcription was performed with 500 ng of total RNA using iScript Reverse Transcription Supermix (Bio-Rad, Hercules, CA, USA) according to the manufacturer's instructions. RT-qPCR analyses comprised three biological replicates per tissue and condition and three technical replicates per biological sample. Reactions were performed in a final volume of 10 μL containing 5 μL of SsoFast EvaGreen Supermix (Bio-Rad), 10 ng of cDNA, and 20 pmol of each primer. Amplification was conducted using a C1000 Thermal Cycler equipped with a CFX384 Touch Real-Time PCR Detection System (Bio-Rad). The thermal program consisted of an initial denaturation at 95 °C for 2 min, followed by 40 cycles of 95 °C for 5 s and 60 °C for 20 s. A melting-curve analysis was then performed from 65 to 95 °C using increments of 0.5 °C every 5 s.

Expression values were normalized using saposin-like aspartyl protease and RNA-binding protein reference genes (Granados et al. 2016). Relative expression was analyzed with the qpcR package using the MAK3 model (Ritz and Spiess 2008). Primer sequences are provided in Supplementary Material 3. Significant differences were determined with a t-test (p-value < 0.05).

S4. Heatmap and correlation analyses

Expression values were represented as $\log_2$ fold changes relative to the corresponding untreated control for each tissue and sampling time. Heatmaps were generated with the pheatmap package (Kolde 2025) in R. Pearson correlation coefficients were calculated from the complete expression dataset (n=54) and visualized using the corrplot package (Wei and Simko 2024) in R. The heatmap displays expression differences (t-test analysis) with a nominal significance threshold of $p < 0.05$, and the correlation plot displays significant Pearson correlations at $p < 0.01$.

References cited in this Supplementary Material

Bouckaert R, Vaughan TG, Barido-Sottani J, Duchêne S, Fourment M, Gavryushkina A, Heled J, Jones G, Kühnert D, De Maio N, Matschiner M, Mendes FK, Müller NF, Ogilvie HA, du Plessis L, Popinga A, Rambaut A, Rasmussen D, Siveroni I, Suchard MA, Wu CH, Xie D, Zhang C, Stadler T, Drummond AJ (2019) BEAST 2.5: An advanced software platform for Bayesian evolutionary analysis. PLoS Comput Biol 15:e1006650. https://doi.org/10.1371/journal.pcbi.1006650

Cañas RA, de la Torre F, Cánovas FM, Cantón FR (2006) High levels of asparagine synthetase in hypocotyls of pine seedlings suggest a role of the enzyme in re-allocation of seed-stored nitrogen. Planta 224:83–95. https://doi.org/10.1007/s00425-005-0196-6

Canales J, Rueda-López M, Craven-Bartle B, Avila C, Cánovas FM (2012) Novel insights into regulation of asparagine synthetase in conifers. Front Plant Sci 3:100. https://doi.org/10.3389/fpls.2012.00100

Clark K, Karsch-Mizrachi I, Lipman DJ, Ostell J, Sayers EW (2016) GenBank. Nucleic Acids Res 44:D67–D72. https://doi.org/10.1093/nar/gkv1276

Corpet F (1988) Multiple sequence alignment with hierarchical clustering. Nucleic Acids Res 16:10881–10890. https://doi.org/10.1093/nar/16.22.10881

Edgar RC (2004) MUSCLE: multiple sequence alignment with high accuracy and high throughput. Nucleic Acids Res 32:1792–1797. https://doi.org/10.1093/nar/gkh340

Goodstein DM, Shu S, Howson R, Neupane R, Hayes RD, Fazo J, Mitros T, Dirks W, Hellsten U, Putnam N, Rokhsar DS (2012) Phytozome: a comparative platform for green plant genomics. Nucleic Acids Res 40:D1178–D1186. https://doi.org/10.1093/nar/gkr944

Granados JM, Ávila C, Cánovas FM, Cañas RA (2016) Selection and testing of reference genes for accurate RT-qPCR in adult needles and seedlings of maritime pine. Tree Genet Genomes 12:60. https://doi.org/10.1007/s11295-016-1018-7

Kolde R (2025) pheatmap: Pretty Heatmaps. R package version 1.0.13. https://doi.org/10.32614/CRAN.package.pheatmap

Kumar S, Stecher G, Suleski M, Sanderford M, Sharma S, Tamura K (2024) MEGA12: Molecular Evolutionary Genetic Analysis version 12 for adaptive and green computing. Mol Biol Evol 41:msae263. https://doi.org/10.1093/molbev/msae263

Ritz C, Spiess AN (2008) qpcR: an R package for sigmoidal model selection in quantitative real-time polymerase chain reaction analysis. Bioinformatics 24:1549–1551. https://doi.org/10.1093/bioinformatics/btn227

Wei T, Simko V (2024) corrplot: Visualization of a Correlation Matrix. R package version 0.95. https://doi.org/10.32614/CRAN.package.corrplot
