# Supplementary material for: Expansion and Nitrate-Responsive Expression of NRT3 Transport Regulators in Maritime Pine
Source: Plants (Basel). 2026 Sep 2;15(17):2690. doi: 10.3390/plants15172690 (PMC13567595; doi:10.3390/plants15172690)
Supplement: Supplementary file 1 [file plants-15-02690-s001.zip › Supplementary_material_S3.pdf]

Supplementary Information
Supplementary Material S3. Primers used for RT-qPCR analysis

Table S1. Complete list of primers used in this study.

Gene	Name	Sequence (5' → 3')	Primer sense
RNA-binding protein	Pp_27526-F	AAGGCTGTCAAACCTGTCCAA	Forward
RNA-binding protein	Pp_27526-R	TTTAGCTATCAGGATGCCTCTG	Reverse
Saposin-like aspartyl protease	Pp_1135-F	AGTATGCTAAGGAATCGTGCCT	Forward
Saposin-like aspartyl protease	Pp_1135-R	GTCCATAATTACACACGAACAGA	Reverse
PpNR	qNR-F	ATCCCCTCCCACATCCTGTTAT	Forward
PpNR	qNR-R	ACAGAGATCAGGAGTGCTCAAT	Reverse
PpNiR	qNiR-F	TTCCAATTCTCATCCATCGCCA	Forward
PpNiR	qNiR-R	TTTCTGAACACGTTGGACTTCG	Reverse
PpNPF1.1	qNPF1.1-F	AGCTGTCATGAGATGGAATGCA	Forward
PpNPF1.1	qNPF1.1-R	GGAGTAGTTACACAGCACACCA	Reverse
PpNPF1.2	qNPF1.2-F	GAGGTGAGTCGAGGTCAGTTTT	Forward
PpNPF1.2	qNPF1.2-R	TTTCGGCAGCAAAATCAGAACC	Reverse
PpNPF1.3	qNPF1.3-F	AAATTCAGAGGTGCGTCAGAGT	Forward
PpNPF1.3	qNPF1.3-R	CCCGCAAAACATAGGACGATTC	Reverse
PpNPF2.1	qNPF2.1-F	CCGACATTACCTCATGCATAGC	Forward
PpNPF2.1	qNPF2.1-R	ACAGGAGTGAGATGTTTTGCCA	Reverse
PpNPF2.2	qNPF2.2-F	GCAGTCTCATTGTCAGCATTGT	Forward
PpNPF2.2	qNPF2.2-R	AATTGAGAGTCCCATATCCCGC	Reverse
PpNPF3.1	qNPF3.1-F	TCCCCACCAACCTCAACAGAG	Forward
PpNPF3.1	qNPF3.1-R	CCACAACGAAAGGCTTGTAGGT	Reverse
PpNPF4.1	qNPF4.1-F	CGAGCACTTCCCCAGATGTATT	Forward
PpNPF4.1	qNPF4.1-R	AGCTACCTAACTAGACTTGCGT	Reverse
PpNPF4.2	qNPF4.2-F	GTGGGTCGTGATATGGTCTGTA	Forward
PpNPF4.2	qNPF4.2-R	TCTGCTTTGTAATCCCTATCACCA	Reverse
PpNPF4.3	qNPF4.3-F	GCTTTTCTATACGCTGCTCTGC	Forward
PpNPF4.3	qNPF4.3-R	TATTTGTACCATCTCGCCCAGG	Reverse
PpNPF4.4	qNPF4.4-F	ACCTTTGTTTGACGTTGTAATCCC	Forward
PpNPF4.4	qNPF4.4-R	AGTACCCAACATCAACGTCACA	Reverse
PpNPF5.1	qNPF5.1-F	GGAGCTTCAGTTGGCATTGGAG	Forward
PpNPF5.1	qNPF5.1-R	TTGAGACGGGCTAGAAGTGGAG	Reverse
PpNPF5.2	qNPF5.2-F	TCTCTGAGTGCAATCAACCTTTGT	Forward

Gene	Name	Sequence (5’ → 3’)	Primer sense
PpNPF5.2	qNPF5.2-R	TGAAGCGAGGAGTGTTCTTTCC	Reverse
PpNPF5.3	qNPF5.3-F	CCCTGGGTATAGCTGTGTATTTGA	Forward
PpNPF5.3	qNPF5.3-R	TTGTCAACGAACCAGCAACTCC	Reverse
PpNPF5.4	qNPF5.4-F	CCTTCGTACATTGGGCACTGTA	Forward
PpNPF5.4	qNPF5.4-R	GACTTTCCCCGAGCAATGCATT	Reverse
PpNPF5.5	qNPF5.5-F	TTCATCGGCATGTTAGTTTGGA	Forward
PpNPF5.5	qNPF5.5-R	TTCCTCTCCATTGCTCCTTCAA	Reverse
PpNPF5.6	qNPF5.6-F	TGCATACACACCAAAGGAAAGCT	Forward
PpNPF5.6	qNPF5.6-R	CAACATACTTGCCCTTGACGCA	Reverse
PpNPF5.7	qNPF5.7-F	AAAAGGCGCCTGATTTTGGTTG	Forward
PpNPF5.7	qNPF5.7-R	GCAGGCTTGGTCTTCTGTTGAG	Reverse
PpNPF5.8	qNPF5.8-F	GCTCATCATCACTTTTACCAGGGT	Forward
PpNPF5.8	qNPF5.8-R	AAACTAAACCTGTCTAGCCCTGA	Reverse
PpNPF5.9	qNPF5.9-F	CGCAGAATAAAATGGCAGTCGT	Forward
PpNPF5.9	qNPF5.9-R	ACTGGCATTCAATTCAGGTACCT	Reverse
PpNPF5.10	qNPF5.10-F	AGGCATTAACAGAGGGCAGAG	Forward
PpNPF5.10	qNPF5.10-R	ACCATCATCAAAAGCCTCTGGG	Reverse
PpNPF5.11	qNPF5.11-F	TGAAATAATTGGTAAGCCCCTCCA	Forward
PpNPF5.11	qNPF5.11-R	TCGCAATACACATACCATATCCCA	Reverse
PpNPF5.12	qNPF5.12-F	TGACATTCACGACCATTTTGCT	Forward
PpNPF5.12	qNPF5.12-R	ACTCCATCATGCATGTCCTGTT	Reverse
PpNPF5.13	qNPF5.13-F	CATGAGCTGCCTGTGGATATCA	Forward
PpNPF5.13	qNPF5.13-R	TAGATTCCTGCATCATCCAGCC	Reverse
PpNPF5.14	qNPF5.14-F	CATTTAGCAAACACGGCCATGT	Forward
PpNPF5.14	qNPF5.14-R	TGTCATCTGTTCGGGAGTTGAG	Reverse
PpNPF5.15	qNPF5.15-F	AGAGGGAAACAACTGAGGCTTT	Forward
PpNPF5.15	qNPF5.15-R	TACTTCCCCATCCTCCTCTCAG	Reverse
PpNPF6.1	qNPF6.1-F	TGAGCTATGCAACCTTCAATCTTG	Forward
PpNPF6.1	qNPF6.1-R	ACGACACTGCCCATTAGACTTT	Reverse
PpNPF6.2	qNPF6.2-F	GGGACACCTAAATATGCAGCTT	Forward
PpNPF6.2	qNPF6.2-R	CTCTGGTCTCCTGCATGTACTC	Reverse
PpNPF6.3	qNPF6.3-F	TCGTATTTTGGGTCGAGGAAGG	Forward
PpNPF6.3	qNPF6.3-R	CCCAAAAGGTATCTCGTTGCTG	Reverse
PpNPF7.1	qNPF7.1-F	TCGTTCGCATGGTTCTGTGTAT	Forward
PpNPF7.1	qNPF7.1-R	GACGATTGGGTGTTCTCTCTGT	Reverse

Gene	Name	Sequence (5' → 3')	Primer sense
PpNPF7.2	qNPF7.2-F	TATGTGCCTGCTTTCCATGAGA	Forward
PpNPF7.2	qNPF7.2-R	GCCAGCGTTGTAAAGAAGTGTT	Reverse
PpNPF7.3	qNPF7.3-F	GACACACAGATTCCATGGCAAC	Forward
PpNPF7.3	qNPF7.3-R	AATCTGTGTGAACTTGGAGCCA	Reverse
PpNPF7.4	qNPF7.4-F	GGCATTCCTTACAGCACTTGAC	Forward
PpNPF7.4	qNPF7.4-R	TTGTCCATGGAACGTGCAATTC	Reverse
PpNPF7.5	qNPF7.5-F	ATGCTTATTACCACCAGAGGCG	Forward
PpNPF7.5	qNPF7.5-R	CTCAATGCAGTCCACAACCAAA	Reverse
PpNPF7.6	qNPF7.6-F	TGTTTCCAAGTTTCAGGGTTGT	Forward
PpNPF7.6	qNPF7.6-R	TCTCTACCTCACACCAAAATAGGA	Reverse
PpNPF7.7	qNPF7.7-F	TCTGTCCAAGGTTAGCAAAGGT	Forward
PpNPF7.7	qNPF7.7-R	TTGACATGATCCAACCATCGCC	Reverse
PpNPF7.8	qNPF7.8-F	CCGTTGTCATTGCACCAAAAGA	Forward
PpNPF7.8	qNPF7.8-R	CGGCCTTTTCTTATGTTATGTCAC	Reverse
PpNPF8.1	qNPF8.1-F	ACACGTTTAGATTTCAGGAGCG	Forward
PpNPF8.1	qNPF8.1-R	CCGAGATCAAACTGCCTCTTCT	Reverse
PpNPF8.2	qNPF8.2-F	ATGCAGCTATCGTAGTGGTCTG	Forward
PpNPF8.2	qNPF8.2-R	CCCAGTTTTGTTAAGCTAGGCA	Reverse
PpNPF8.3	qNPF8.3-F	GTATGGATCTCAACATTGCGCC	Forward
PpNPF8.3	qNPF8.3-R	CAAGCTGAAAACGCACAAGTCT	Reverse
PpNPF8.4	qPpNPF8.4-F	CCTAGAAATTGCTGGAGAACAGA	Forward
PpNPF8.4	qPpNPF8.4-R	TAATCTGGCCAATCCTGAACAAC	Reverse
PpNRT3.1	qNRT3.1-F	CAGTTTGCAAGTGTCTAGAACGG	Forward
PpNRT3.1	qNRT3.1-R	CCTGTCAAGCCCACTACAATCT	Reverse
PpNRT3.2	qNRT3.2-F	TTAGATGCAGAAGGAGCCCAAA	Forward
PpNRT3.2	qNRT3.2-R	AATGAGACTGGATGCGGGATAC	Reverse
PpNRT3.3	qNRT3.3-F	GCTGTCATTTCTTACCTCAACCA	Forward
PpNRT3.3	qNRT3.3-R	CGGTCCTTTTCAAGATTGTCGG	Reverse
PpNRT3.4	qNRT3.4-F	AGTGAACAGGGAGAATGCAAATG	Forward
PpNRT3.4	qNRT3.4-R	ATACAGGACTCCACCCCTAAGT	Reverse
PpNRT3.5	qNRT3.5-F	TTTCCCTGGACATCATCACTGC	Forward
PpNRT3.5	qNRT3.5-R	TTAATTCTTCTGCGATGCCCTG	Reverse
PpNRT2.1	qNRT2.1-F	AGACCAAGGAGAACGGATACAA	Forward
PpNRT2.1	qNRT2.1-R	CATTGAACGCTTGCTCTGATTT	Reverse
PpNRT2.2	qNRT2.2-F	GCTAATCGTGCTAATATGCCGC	Forward

Gene	Name	Sequence (5’ → 3’)	Primer sense
PpNRT2.2	qNRT2.2-R	TTGTTGAGCCTCTTACTTGCAG	Reverse
