# Supplementary material for: Expansion and Nitrate-Responsive Expression of NRT3 Transport Regulators in Maritime Pine
Source: Plants (Basel). 2026 Sep 2;15(17):2690. doi: 10.3390/plants15172690 (PMC13567595; doi:10.3390/plants15172690)
Supplement: Supplementary file 1 [file plants-15-02690-s001.zip › Supplementary_material_S5.pdf]

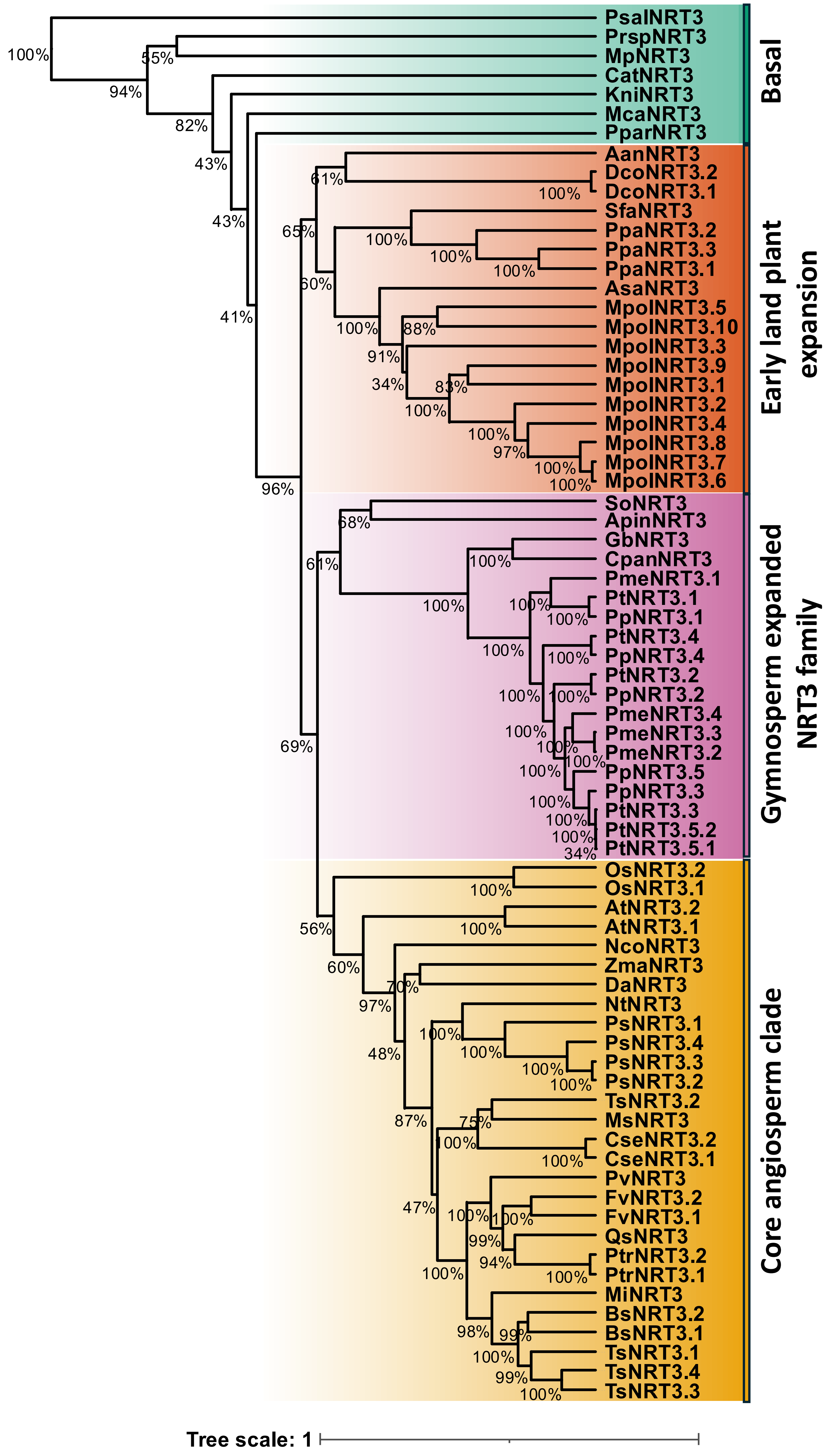

Fig. S1. Bayesian phylogenetic analysis of the plant *NRT3* nucleotide sequences. The maximum clade credibility tree was inferred from *NRT3* nucleotide sequences using BEAST2 with the GTR substitution model with gamma-distributed rate heterogeneity, a strict molecular clock and a Yule tree prior. *Picocystis salinarum* was used as the outgroup. Node values indicate Bayesian posterior probabilities expressed as percentages. Major *NRT3* groups are highlighted according to their phylogenetic distribution, including early-diverging/basal homologs, early land plant-expanded lineages, gymnosperm/conifer-expanded *NRT3* paralogs and angiosperm-associated clades.

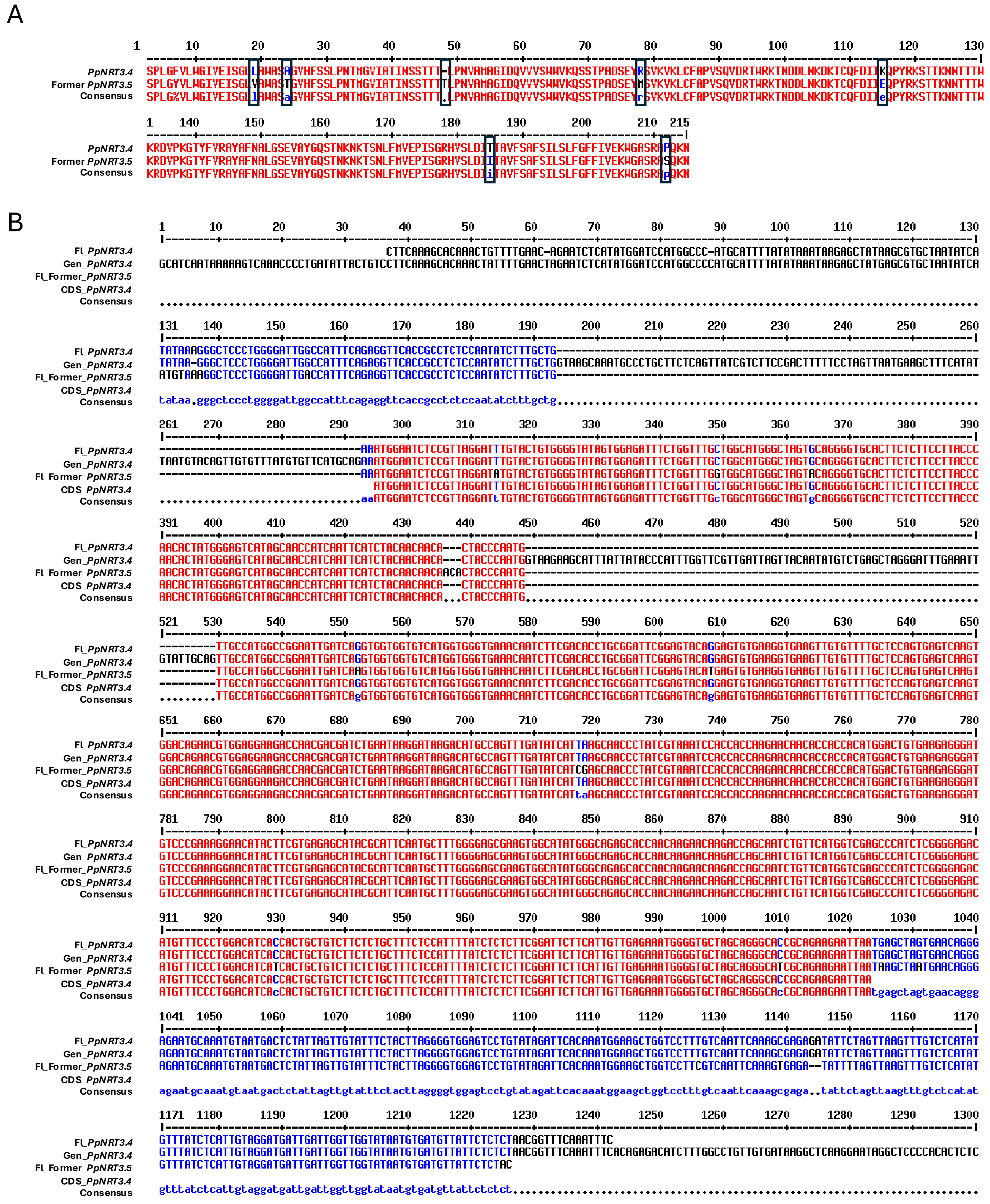

Fig S2. Sequence comparison between PpNRT3.4 and the sequence formerly designated as PpNRT3.5. (A) Pairwise alignment of the predicted amino acid sequences. Divergent residues are enclosed in boxes. (B) Nucleotide alignment of the full-length sequences of *PpNRT3.4* (FL_*PpNRT3.4*) and the former *PpNRT3.5* (FL_Former_*PpNRT3.5*) with the genomic (Gen_*PpNRT3.4*) and coding sequences (CDS_*PpNRT3.4*) of *PpNRT3.4*. Coding regions, untranslated regions, and intronic or flanking genomic regions are shown in red, blue, and black, respectively. Dashes indicate alignment gaps. The high overall sequence conservation and limited number of amino acid substitutions support the reassignment of the former *PpNRT3.5* sequence as an allelic variant of *PpNRT3.4*, rather than as a distinct paralog.
